# Evaluation of Anti-Müllerian Hormone (AMH) Serum Levels in Patients with Polycystic Ovary Syndrome (PCOS) Depending on Body Mass Index (BMI)

**DOI:** 10.3390/jcm14082677

**Published:** 2025-04-14

**Authors:** Amalia Gorzko, Mariia Melnyk, Jolanta Nawrocka-Rutkowska, Andrzej Starczewski, Aleksandra Marciniak, Iwona Szydłowska

**Affiliations:** 1 Individual Medical Practice, 71-276 Szczecin, Poland; amaliagorzko@op.pl; 2 Department of Gynecology, Endocrinology and Gynecological Oncology, Pomeranian Medical University in Szczecin, 71-252 Szczecin, Poland; marichkakopot@gmail.com (M.M.); jolanta.nawrocka.rutkowska@pum.edu.pl (J.N.-R.); andrzej.starczewski@pum.edu.pl (A.S.); o.marciniak@wp.pl (A.M.)

**Keywords:** PCOS, BMI, AMH, obesity

## Abstract

The relationship between anti-Müllerian hormone (AMH) levels and body weight, expressed through body mass index (BMI), in women with PCOS has been a topic of discussion for a long time, yet the literature continues to present conflicting data. The latest guidelines emphasize the growing role of AMH in the diagnosis of polycystic ovary syndrome and suggest that it should become one of the diagnostic criteria for identifying this condition. **Objectives**: The aim of this study was to determine the relationship between AMH levels and BMI in reproductive-age patients. The bioethics committee approved the conduct of the study. **Methods:** A total of 193 patients diagnosed with polycystic ovary syndrome (PCOS) based on the Rotterdam criteria were included in the study group. The control group consisted of 196 patients who did not meet the diagnostic criteria for PCOS. Blood samples (5 mL of venous blood) were collected from all participants to determine AMH levels. Additionally, body weight and height were measured, and BMI was calculated. **Results**: The mean AMH level for women with PCOS was 7.187 ng/mL (median: 6.400 ng/mL) and was more than twice as high as women without PCOS (mean: 3.399 ng/mL, median: 2.835 ng/mL). The decline in the average AMH level occurs at an older age (35–39 years) in women with PCOS compared to women without PCOS (25–29 years). A significant negative correlation between AMH levels and BMI was observed only in women with PCOS in the 25–29 age group. In women in the 20–24 age group, this correlation takes the form of a statistical tendency. **Conclusions:** Obesity is a modifiable factor influencing AMH levels. The demonstrated relationship between AMH and BMI may contribute to the development of therapeutic protocols tailored to the patient’s clinical condition.

## 1. Introduction

Polycystic ovary syndrome (PCOS) is one of the most common multidisciplinary disorders occurring in women of reproductive age. This condition affects as many as 10–15% of women in their reproductive years [1]. The symptoms and their severity vary among individuals, and the long-term health risks remain a therapeutic challenge not only for gynecologists but also for other medical specialists, such as endocrinologists, psychiatrists, and dietitians [2]. Accordingly, therapeutic decisions must be individualized and made with consideration of potential comorbidities associated with PCOS, such as obesity, metabolic abnormalities (primarily related to glucose metabolism), and infertility in order not only to address health-related issues but also to improve patients’ quality of life [1].

The diagnosis of polycystic ovary syndrome (PCOS) has been based on the criteria established in 2003 by ESHRE in Rotterdam [1]. These criteria require the presence of two out of three clinical signs: ovulatory dysfunction in the form of oligo- or anovulation, clinical and/or laboratory signs of hyperandrogenism, and the characteristic polycystic ovarian morphology (PCOM) seen in ultrasound examination. However, it must be stated that PCOS is a diagnosis of exclusion—before diagnosing it, other conditions such as congenital adrenal hyperplasia, Cushing’s syndrome, hypothyroidism or androgen-secreting tumors must be excluded [1,2,3]. Currently, anti-Müllerian hormone (AMH) is gaining an increasingly important role in the diagnosis of PCOS. AMH is a glycoprotein belonging to the transforming growth factor-beta family, produced and released by granulosa cells in preantral and antral ovarian follicles [2]. It is considered a marker of ovarian reserve, as it plays a role in the maturation and selection of follicles. As serum AMH levels correlate with the number of growing follicles in the ovaries, this hormone is considered an indicator of both the quantity and quality of a woman’s ovarian reserve [4].

In women, AMH levels decline with age and become undetectable after menopause. It is assumed that peak levels of this hormone are reached by women between the ages of 21 and 30, on average, at 24.5 years of age [5]. During reproductive age, patients with PCOS have approximately two to three times higher serum AMH levels compared to individuals without the condition [5,6]. This is particularly significant for women with polycystic ovary syndrome who are unsuccessfully trying to conceive.

AMH levels are influenced by many factors [3]. According to most sources, the use of hormonal contraception lowers the levels of this hormone [7], and smoking has a similar effect [8]. Ethnic factors also influence AMH levels, which is why it is crucial to assess their values in specific populations [9,10].

The latest guidelines propose a modification of the Rotterdam criteria, suggesting that (AMH) may be used interchangeably with polycystic ovarian morphology (PCOM) as a diagnostic criterion for the diagnosis of PCOS in adults [1]. Ultrasonographic examination is recognized to have certain limitations: it is dependent on the operator’s expertise, the sensitivity of the equipment used, the patient’s body mass, and the examination approach (transvaginal or transabdominal). While the transabdominal method may be more acceptable to some patients—due to cultural considerations or sexual status—it is generally regarded as less accurate [1]. In contrast, serum AMH levels appear to circumvent the aforementioned sources of subjectivity.

Despite the increasing role of AMH in diagnostic algorithms for PCOS and treatment strategies for this condition, the literature presents conflicting data regarding the correlation between AMH levels and factors such as obesity [4,11].

Obesity is becoming an increasingly serious challenge on a population level which impacts the overall health of society. Obesity societies refer to this condition as a “pandemic”, and future forecasts suggest that the problem will worsen [12]. In women with PCOS, abdominal obesity is particularly common and is associated with a higher number of health complications [12]. Excessive fat tissue located in the abdominal area is connected to insulin resistance and exacerbates metabolic disorders in patients, worsening fertility issues and influencing the expression of clinical symptoms [13]. Therefore, most recommendations advocate weight loss as the first step in treatment.

The screening test for diagnosing obesity in adults is the measurement of body weight and height, followed by the calculation of BMI (body mass index) [12]. BMI is considered a standardized indicator for assessing nutritional status. If its value exceeds the normal range, it indicates whether the individual is overweight (BMI 25.0–29.9 kg/m^2^) or obese (BMI ≥ 30 kg/m^2^).

Because obesity is, as emphasized earlier, often associated with PCOS, it becomes even more crucial to determine its correlation with a potential diagnostic factor of PCOS, such as AMH levels. Studies on this topic have been conducted for other populations [4,9,10], which is why we believe that this study will fill a gap in the context of the Central European population.

Because laboratories also provide quite broad reference ranges for AMH levels in women with PCOS, regardless of their age, our aim was also to establish these reference values. The laboratory where this study was conducted uses AMH reference values ranging from 1.86 to 18.90 ng/mL, with a median value of 6.81 ng/mL.

## 2. Materials and Methods

### 2.1. Study Group

This study is a retrospective-prospective study. It includes patients aged 18–49 years. Prior to participation, all patients were provided with written information detailing the study’s objectives and procedures. They signed an informed consent form and granted permission for the processing of their personal data in compliance with the European Parliament and Council Regulation (EU 2016/679 of 27 April 2016 on the protection of individuals with regard to the processing of personal data, Official Journal of the European Union L 119 from 4 May 2016, p. 1).

The study was approved by the Bioethics Committee of the Pomeranian Medical University (approval number KB-006/12/2024 on 13 March 2024). The study group comprised 193 patients diagnosed with polycystic ovary syndrome (PCOS) according to the Rotterdam criteria. Meanwhile, the control group included 196 patients who did not fulfill the diagnostic criteria for PCOS.

A detailed medical history was collected from all participants regarding their general health status. Individuals with concurrent systemic diseases (type I and II diabetes, hypertension, heart diseases, liver diseases) that could affect the study results were excluded from participation. Patients had their hormonal medications discontinued for at least 3 months before the treatment.

All patients underwent a gynecological examination and ultrasound (transvaginal) to assess the reproductive organs, with particular attention given to the ovaries, including evaluation of their volume, as well as the number, diameter, and distribution of follicles. Ultrasound pictures of ovaries were assessed using an Alpinion X-CUBE 70 imaging system; 11 Mhz endovaginal transducer.

Body weight and height measurements were taken from the patients and based on these measurements, the BMI (body mass index) was calculated (body mass index = body weight in kg/height in m^2^). The interpretation of BMI values was adopted according to the WHO criteria as follows: underweight, normal weight, overweight, and obesity [14].

From the perspective of the study objectives, the diverse age structure of the examined groups made direct comparisons challenging. Therefore, it was justified to conduct analyses within the following age groups: 18–19 years, 20–24 years, 25–29 years, 30–34 years, 35–39 years, and 40 years and older.

### 2.2. Biochemical Tests

Serum samples (5 mL of venous blood) were obtained on days 3–5 (early follicular phase) of the menstrual cycle. Laboratory tests were performed at the same laboratory. Roche kits were used for the measurements. AMH was measured using the Elecsys test and the ECLIA method on a Cobas device [15].

### 2.3. Statistical Analysis

Statistical calculations were performed using the Statistica v. 13.1 software package. The normality of distributions was tested using the Kolmogorov–Smirnov and Shapiro–Wilk tests. The homogeneity of variances was tested using Levene’s test. Means were compared using Welch’s *t*-test, medians were compared using the Mann–Whitney U test, and differences in proportions (structural indices) were analyzed using the z-test for two proportions. Relationships between quantitative variables were described using the Pearson linear correlation coefficient and/or the Spearman rank correlation coefficient.

The significance level was set at *p* < 0.05. A statistical tendency was described in cases where the *p*-value was close to 0.05.

## 3. Results

The characteristics of the study and control groups are presented in Table 1. Compared to the control group, patients in the study group were notably younger (*p* < 0.001), had higher BMI values (*p* < 0.001) that fell in all age categories within the overweight range, and exhibited significantly elevated AMH levels (*p* < 0.001).

**Figure 1:**
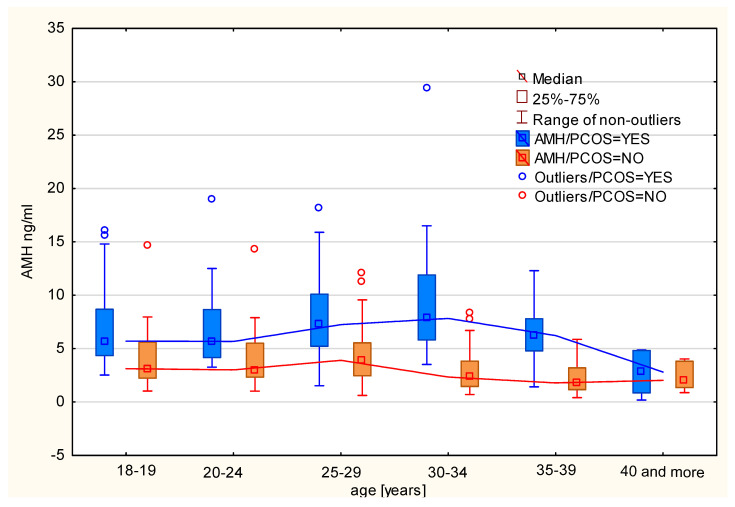
Median AMH levels by age category in women with and without PCOS.

The mean and median AMH levels for women with polycystic ovary syndrome in the Polish population were estimated. It was noted that patients with PCOS had AMH levels more than twice as high (mean: 7.187 ng/mL, median: 6.400 ng/mL) as women without PCOS (mean: 3.399 ng/mL, median: 2.835 ng/mL). After dividing the patients into age categories, it was observed that the arithmetic mean of AMH levels for patients without PCOS in different age categories follows the model by Kelsey et al. [16]. An increase in AMH levels in the subsequent age categories was observed up to the 25–29 age range, followed by a decline. Among patients with PCOS, the average (arithmetic mean and median) AMH levels are significantly higher compared to healthy women in each age category. These data are presented in Figure 1 and Table 2 and indicate that a decline in the average AMH levels occurs in the older age group (35–39 years) compared to women without PCOS. Moreover, the variability of AMH levels is greater among patients with PCOS than among those without PCOS in all age categories.

**Figure 2:**
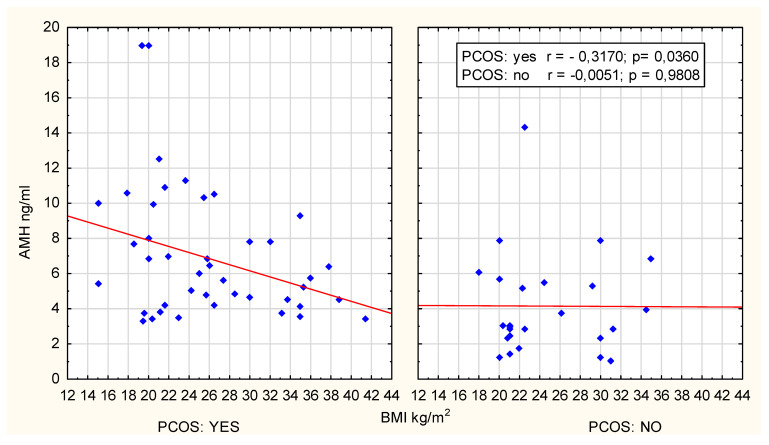
Scatter plot; age group 20–24 years.

After dividing the patients into age categories, a correlation analysis was conducted between AMH concentration and BMI in different groups of women with PCOS. In the case of women in the 18–19 age group, no correlation was observed between AMH and BMI (r = 0.0224; *p* = 0.8685). In the 20–24 age group, a negative correlation between these parameters was identified (r = −0.3170, *p* = 0.036). These data are graphically presented in Figure 2.

However, excluding two extreme outliers from the analysis renders the correlation statistically insignificant (r = −0.2557, *p* = 0.1021) and using Spearman’s rank correlation (R = −0.230, *p* = 0.134) confirmed the above observation, establishing it as a statistical tendency.

**Figure 3:**
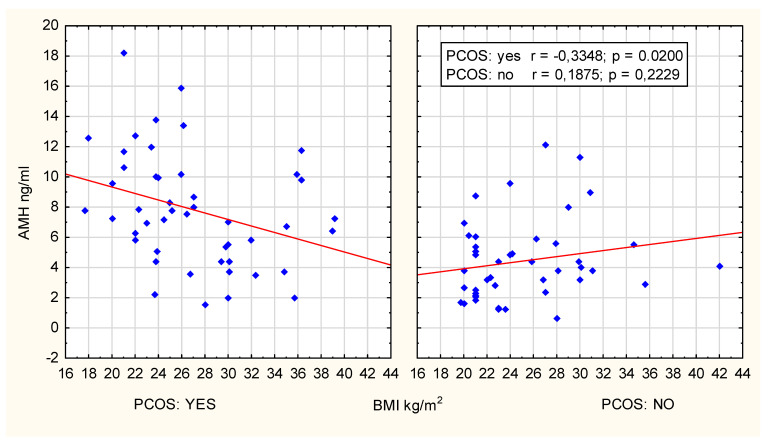
Scatter plot; age group 25–29 years.

A clear and statistically significant negative correlation between AMH concentration and BMI was observed in women with PCOS aged 25–29 years old (r = −0.3348; *p* = 0.02, R Spearman’s = −0.391; *p* = 0.006), as presented in Figure 3.

The group of women aged 30–34 years old was too small in size to conduct a statistical correlation analysis. Among women aged 35 and older with PCOS, no statistically significant relationship was found between the variables studied.

In the control group, no correlations were observed.

## 4. Discussion

Polycystic ovary syndrome is a very common endocrine disorder in women of reproductive age. As a condition, it has a very heterogeneous presentation, with a wide range of clinical symptoms and varying degrees of severity. The symptoms of the disease are associated with hormonal, metabolic, and reproductive disorders, as well as affecting the mental health sphere [1]. The prevalence of this disease suggests the need for a multifaceted approach to address its diversity. Obesity is also becoming an increasingly serious challenge on a population level and impacts the overall health condition of society [12].

It is often believed that the use of anti-Müllerian hormone instead of the ultrasonographic criterion allows for the diagnosis of a greater number of women who would not have been diagnosed with polycystic ovary syndrome if only the Rotterdam criteria were used [1]. AMH is an important and very sensitive indicator of ovarian reserve and can be further used to predict ovarian response to hormonal stimulation and assess the risk of ovarian hyperstimulation syndrome as a complication of ovulation induction in women with PCOS [1,2]. Therefore, its diagnostic role, especially in women of reproductive age trying to conceive, cannot be overestimated.

Despite the increasing importance of AMH in diagnostic algorithms for PCOS and in the treatment approaches for this condition, the literature presents conflicting data regarding the correlation between the values of this hormone and factors such as obesity or the ethnic origin of the patients [4,5,9,11].

To the best of our knowledge, reference ranges for AMH have not been developed for the Polish population, from which our patients were recruited, and the reference ranges used in laboratories are very broad. Therefore, we decided to examine this in the studied population.

The mean and median values of AMH for the population of women with PCOS are also reflected in the literature. We observed that the mean and median AMH concentrations in women with polycystic ovary syndrome are almost twice as high as those achieved by women in the control group. Similar to the values obtained in this study, Butt et al. found comparable mean AMH concentrations in women with PCOS in the Pakistani population (mean AMH value of 7.23 ng/mL) [17], and Zadehmodarres et al. reported a mean AMH value of 7.14 ng/mL for women with PCOS in the Iranian population [18]. However, these values should be approached with caution, as they were derived from populations different from the European one.

Many researchers emphasize the negative correlation between AMH concentrations and BMI values that was presented in this study. Ou et al. note that in the Chinese population, there is a negative relationship between anti-Müllerian hormone levels and BMI and because of this observation, they conclude that AMH could be a good predictor of metabolic disorders in women with polycystic ovary syndrome [19]. The same issue for the Chinese population was addressed by Han Zhao et al., who also found a negative correlation between AMH concentration and BMI in women with PCOS [20]. This team also investigated the relationship between anti-Müllerian hormone levels and the presence of insulin resistance, a topic that we did not cover in this preliminary report, but we intend to conduct further research in this area. A strong negative correlation between AMH concentration and BMI in women with PCOS is reported by Kloos et al. [21]. This study also included individuals with morbid obesity and a BMI > 45, a group that we were unable to sufficiently represent in our study because the sample was predominantly composed of overweight patients. The similar distribution of body weight may, however, stem from differences between populations. A negative correlation between BMI and AMH concentration was also observed by Güngör et al. in the Turkish population of women with PCOS [22]. In our study, a significant negative correlation between AMH levels and BMI for Polish women with PCOS was observed only in the 25–29 age group. In the 20–24 years age group, this correlation is characterized only as a statistical tendency and for other age groups, we did not observe such a correlation. Although our study did not show this for the control group, there are studies that have reported a negative correlation between AMH concentration and BMI in both women with PCOS and healthy women. Wang et al., citing a study conducted by Moy et al., confirm that obese Caucasian women, regardless of the presence of polycystic ovary syndrome, exhibit lower AMH levels than their lean counterparts [23]. However, it is possible that other factors not analyzed in this study may have a lowering effect on AMH levels in healthy women.

The results of our study contrast with those obtained by researchers such as Casadei et al., who, studying a population of infertile women both with and without PCOS, found no correlation between AMH concentration and BMI [24]. Similar conclusions were drawn by the German research team led by Gorkem et al., who reported no correlation between AMH and BMI [25]. Goueva et al. also found no correlation between the potential presence of obesity and AMH concentrations. However, it is important to note that their study only included individuals of perimenopausal age, unlike in our study, where the patient group was diverse in terms of age [26]. Contradictory results were observed by Bahadur A et al., who found a weak positive, but statistically insignificant, correlation between BMI and AMH in the Indian population [27].

An interesting clinical observation was made by an Italian group led by Meneghini et al. They found that overweight and obesity are significant risk factors for adverse effects during hormone stimulation and a reduced response to ovulation induction. After participants followed a very-low-calorie diet for 120 days, the researchers noted not only a significant decrease in BMI but also a reduction in antral follicle count (AFC) and AMH levels. This led to a statistically lower incidence of ovarian hyperstimulation syndrome (OHSS). The authors concluded that weight reduction may improve reproductive health, restore menstrual regularity, and reduce the risk of OHSS in women with PCOS undergoing artificial reproductive techniques (ART) [28].

In summary, our study determined AMH levels in women with PCOS in the Central European population, categorized by age. Following other scientific publications, our research confirms a significant negative correlation between AMH levels and BMI in women with PCOS only in the 25–29 age group. In the 20–24 age group, this correlation is characterized as a statistical tendency.

### Strengths and Limitations

The strength of this study lies in its sample size, which is even more valuable taking the detailed inclusion and exclusion criteria for the study into consideration. However, it would certainly be valuable to include more representatives in the 30–34 age group, which was underrepresented in the study sample. Nevertheless, it should be emphasized that the relationship between AMH concentrations and BMI is not necessarily a simple one. Other factors not considered in this study, such as the type of obesity characterized by fat tissue distribution in the body or the presence of insulin resistance, may influence the conclusions drawn here. Therefore, these findings should be interpreted with caution. There are plans to include the aforementioned factors as part of future research and we believe that the results obtained in this way will expand our knowledge about the role of AMH in PCOS.

## 5. Conclusions

There is an increasing call to modify the diagnostic criteria of PCOS. The 2023 recommendations of the European Society of Human Reproduction and Embryology (ESHRE) suggest that serum anti-Müllerian hormone (AMH) levels could serve as an alternative to ultrasound examination in diagnosing PCOS [1]. At the same time, ESHRE criteria emphasize the importance of using a population-specific cutoff value in AMH measurements. Laboratories adopt a very wide range of reference values for AMH, ranging from 1.86 to 18.90 ng/mL for women with PCOS. Therefore, we decided to specify the AMH values for women with polycystic ovary syndrome in the Central European population across different age groups. We have correlated them with BMI values to determine whether overweight/obesity influence AMH in these patients. This is very important for reproductive-age women in their preparation for pregnancy. This study demonstrated a significant negative correlation between AMH levels and BMI, observed only in women with PCOS in the 25–29 age group, while such a correlation in the 20–24 age group should be considered as a statistical tendency.

This finding appears particularly significant given the importance of identifying factors influencing AMH concentration as a potential diagnostic criterion for PCOS and as a factor aiding in the formulation of therapeutic strategies for the disease. We plan to expand our research in the future to include other factors, such as the localization of adipose tissue in the body, that may influence AMH.

## Figures and Tables

**Table 1:** Characteristics of the groups.

Parameters		PCOS Group	Non-PCOS Group	*p* Value
age [years]	mean	25.16	29.29	*p* < 0.001 ^(1)^
	standard deviation	6.878	6.816	NSS *
	median	24.00	30.00	*p* < 0.001 ^(3)^
BMI [kg/m^2]^	mean	26.96	24.07	*p* < 0.001 ^(4)^
	standard deviation	6.092	5.165	*p* < 0.001 ^(2)^
	median	26.00	22.32	*p* < 0.001 ^(5)^
AMH [ng/mL]	mean	7.187	3.399	*p* < 0.001 ^(6)^
	standard deviation	3.957	2.447	*p* < 0.001 ^(2)^
	median	6.400	2.835	*p* < 0.001 ^(7)^

* NSS = not statistically significant. ^(1)^ t = −5.948, df = 386.77—Welch’s test; ^(2)^ Levene test ^(3)^ U = 12,340.5; z = −5.928—Mann–Whitney test; ^(4)^ t = 0.5043; df = 375.01—Welch’s test; ^(5)^ U = 13,137; z = 5.210—Mann–Whitney test; ^(6)^ t = 11.335; df = 319.25—Welch’s test; ^(7)^ U = 6497; z = 11.198—Mann–Whitney test.

**Table 2:** Comparison of the studied populations with AMH and BMI values in different age groups.

		18–19 Years			20–24 Years			25–29 Years		
		PCOS	NON-PCOS	*p*	PCOS	NON-PCOS	*p*	PCOS	NON-PCOS	*p*
Sample size (n)		57	26		44	25		48	44	
BMI [kg/m^2^]	mean	26.17	20.55	*p* < 0.001	25.81	24.59	NSS	27.29	25.05	*p* < 0.05
	median	24.85	20.00	*p* < 0.001	25.23	22.27	NSS	26.10	23.29	*p* < 0.05
	standard deviation	5.78	3.65	*p* < 0.05	6.81	5.11	NSS	5.79	4.99	NSS
AMH [ng/mL]	mean	6.88	4.17	*p* < 0.001	6.88	4.15	*p* < 0.001	7.76	4.42	*p* < 0.001
	median	5.70	3.13	*p* < 0.001	5.68	3.01	*p* < 0.001	7.25	3.91	*p* < 0.001
	standard deviation	3.46	3.01	NSS	3.73	2.94	NSS	3.73	2.68	*p* < 0.05
		30–34 years			35–39 years			40 and more		
		PCOS	NON-PCOS	*p*	PCOS	NON-PCOS	*p*	PCOS	NON-PCOS	***p***
Sample size (n)		17	47		22	42		5	12	
BMI [kg/m^2^]	mean	28.86	23.11	*p* < 0.001	29.20	25.25	*p* < 0.05	26.69	26.61	NSS
	median	30.00	22.10	*p* < 0.001	29.88	22.96	*p* < 0.05	25.90	24.22	NSS
	standard deviation	5.54	3.13	*p* < 0.05	6.49	6.23	NSS	3.11	7.28	NSS
AMH [ng/mL]	mean	9.71	2.89	*p* < 0.001	6.41	2.26	*p* < 0.001	2.71	2.38	NSS
	median	7.83	2.34	*p* < 0.001	6.23	1.79	*p* < 0.001	2.81	2.03	NSS
	standard deviation	6.34	1.84	*p* < 0.001	2.91	1.55	*p* < 0.05	2.18	1.24	NSS

NSS—not statistically significant.

## Data Availability

The original contributions presented in this study are included in the article. Further inquiries can be directed to the corresponding author.
